# Collaboration to Increase Colorectal Cancer Screening Among Low-Income Uninsured Patients

**Published:** 2011-04-15

**Authors:** Diana Redwood, Larry Holman, Sharon Zandman-Zeman, Tom Hunt, Leah Besh, Wanda Katinszky

**Affiliations:** Ride for Life Alaska; Ride for Life Alaska, Anchorage, Alaska; Anchorage Neighborhood Health Center, Anchorage, Alaska; Anchorage Neighborhood Health Center, Anchorage, Alaska; Anchorage Neighborhood Health Center, Anchorage, Alaska; Providence Alaska Medical Center, Anchorage, Alaska

## Abstract

**Background:**

Colorectal cancer (CRC) is the second leading cause of cancer-related deaths in the United States. CRC screening allows for prevention through the removal of precancerous lesions and early detection of cancer.

**Community Context:**

Ride for Life Alaska (RFL), a nonprofit organization that raises funds to fight cancer, and the Anchorage Neighborhood Health Center (ANHC), which is Alaska's largest community health center, joined efforts to provide CRC screening and outreach to an ethnically diverse group of low-income underinsured or uninsured patients residing in and around Anchorage, Alaska.

**Methods:**

RFL and ANHC worked with gastroenterologists, medical practices, and pathology services to contribute pro bono and reduced-fee services for CRC screening. Information to patients was distributed through signs in the clinic, flyers, and the ANHC website.

**Outcomes:**

CRC screening was increased in this population. During 2007-2009, there were 2,561 immunochemical fecal occult blood tests given to patients, and 1,558 were completed (61%); 24% were positive. Sixteen gastroenterologists, 4 medical practices, and 2 laboratories provided 111 follow-up colonoscopies and pathology services to patients identified through the CRC screening program who did not have other funding resources available for follow-up care.

**Interpretation:**

This program provides a model for leveraging scarce screening resources by drawing on multiple partners to increase CRC screening. Recommendations for those seeking to initiate similar programs are to have memoranda of agreement in place and a clear scope of work for all participating people and organizations to avoid delays in program implementation; hire a screening care coordinator to manage patient care and collaborate with medical practices; and identify program champions who have the energy and persistence to craft such partnerships.

## Background

Colorectal cancer (CRC) is the second leading cause of cancer-related deaths in the United States. In 2009, an estimated 150,000 people had CRC diagnosed and 50,000 died from the disease (1). CRCs can develop from slow-growing adenomatous polyps in the colon and rectum. Screening for CRC allows the identification and removal of precancerous lesions and early detection of cancers (2-4). Various screening modalities reduce CRC deaths. Annual fecal occult blood tests (FOBTs) or colonoscopy every 10 years are both CRC screening tests recommended by the US Preventive Services Task Force (USPSTF) (4). However, screening for this disease is underused, especially among people with low income, the underinsured and uninsured, and minorities (5,6). Some reasons for low screening rates include confusion regarding which test to perform of the many options available, lack of awareness of the need for screening or fear of screening among potential patients, and cost and capacity issues (7-9).

## Community Context

We describe the development, implementation, and outcome of a partnership between a fund-raising organization, Ride for Life Alaska (RFL), and a community health center, the Anchorage Neighborhood Health Center (ANHC). The objective of the partnership is to increase the number of CRC screenings completed by low-income underinsured and uninsured patients. To provide more screenings, the partnership engages the medical community: primary care providers and specialty care providers, including gastroenterologists, medical practices, and pathology services.

ANHC began providing primary care services in 1971 and has become 1 of Alaska's largest primary care medical practices. ANHC revenue comes from the following sources: Medicaid/Medicare (56%), private insurance (25%), and self-pay by patients (19%). However, grants make up a substantial portion (38%) of the total operating budget. ANHC is a federally qualified health center and is Alaska's largest community health center, serving 11,500 patients through 40,000 visits per year. More than half of patients (58%) are at or below the federal poverty level, and only 18% of patients have private insurance. The patient population is ethnically diverse, and more than half (59%) report a nonwhite ethnicity. Asians/Pacific Islanders make up 20% of patients, followed by Hispanics/Latinos (12%), African Americans (12%), and Alaska Natives (2%). There is also a diversity of languages spoken by patients, including Spanish, Samoan, Korean, and Hmong. The population served has more women (56%) than men (44%), and 46% are aged 45 years or older.

**Figure 1:**
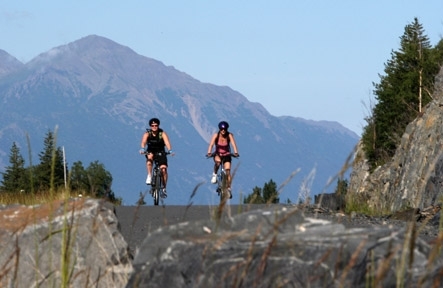
Ride for Life Alaska fundraiser bicycle participants, 2007

RFL was founded in 2004 by Anchorage resident Larry Holman after he was diagnosed with stage 3 CRC. While in treatment, Holman and several friends and acquaintances began planning a 2-day, 125-mile bicycle ride through 2 scenic mountain passes from Anchorage to Seward, Alaska (Figure 1). Originally intended as a way for Holman to celebrate and appreciate his life, friends, and family, the ride turned into a cancer fundraiser. Holman, having just experienced the personal effects of CRC, felt passionately about raising funds for cancer prevention and education. A core group of volunteers joined efforts to move the project forward. Event coordination included gathering all necessary permits and insurance, advertising with bicycle clubs and outdoor groups, and setting up camping space, food, rest stops, support stations, and transportation for participants back to Anchorage after the ride. The first night of the ride also includes a "Celebration of Life" dinner, which features speakers and riders sharing their cancer experiences and honoring those who have died of cancer.

The first ride in August 2004 was a success and became an annual ride that grossed $120,000 in cash and in-kind contributions in 2009. RFL incorporated as a 501(c) nonprofit in December 2004. For the first 2 years, RFL donated funds raised to the Lance Armstrong Foundation Peloton Project. The Lance Armstrong Foundation had good name recognition for riders, a mechanism in place to donate funds via the Internet, and low administrative costs compared with other nonprofit groups.

In 2006, the state of Alaska and the Alaska Tribal Comprehensive Cancer Control Programs contacted RFL and suggested that the money raised be used locally to fund a CRC screening program for Alaska residents instead of directed solely toward the national Peloton Project. RFL contacted ANHC to explore setting up a partnership to promote CRC screening among low-income and underinsured ANHC patients. Before this partnership, there were no community-based CRC screening services available for low-income patients. ANHC offered $10 FOBT kits to patients on request, but the cost was still a barrier to the neediest patients. Before initiation of the partnership, only about 200 kits were distributed each year. RFL and ANHC signed a memorandum of agreement in 2007. This type of collaboration between a fundraising organization and a health facility had never been attempted in Alaska before. Additionally, although ANHC had patients in need of screening, it did not have the capacity to provide all the elements necessary for a comprehensive screening program, including follow-up for positive screening exams.

## Methods

### Engaging the medical community

In 2006, RFL and ANHC invited members of the Anchorage medical community to participate in the initiative to increase CRC screening among the ANHC patient population. Staff from the 2 organizations called, sent letters, and e-mailed the 16 gastroenterologists working in the Anchorage area. All Anchorage gastroenterologists in private practice agreed to provide pro bono colonoscopies. The private endoscopy centers in which the endoscopy providers worked agreed to provide discounted facility fees. The laboratories that processed their specimens then agreed to provide discounted pathology services. This initial phase of engaging the medical community took about a year to set up memoranda of agreement among all the parties involved.

**Figure 2:**
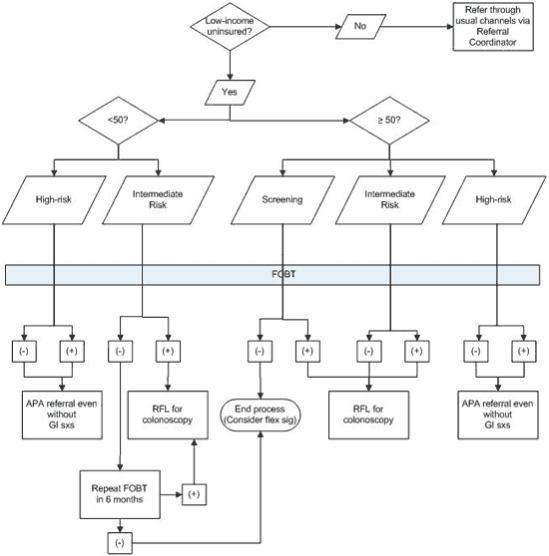
Referral flow chart, Anchorage Neighborhood Health Center, 2009. Note: FOBT should be performed on all referrals. A negative test does not necessarily deflect referral when appropriate but is helpful collateral information. Definitions: intermediate risk, no specific gastrointestinal symptoms and either family history or weight loss; high-risk, a person with a history of colon cancer, first degree family history, or change in stool pattern and rectal bleeding or unexplained weight loss. Abbreviations: FOBT, fecal occult blood test; APA, Anchorage Project Access; RFL, Ride for Life Alaska funding; flex sig, flexible sigmoidoscopy; GI, gastrointestinal; sxs, symptoms.

Key to engaging the private gastroenterologists was the development of mutually agreeable criteria for referral. ANHC developed a flow chart (Figure 2) designed to keep the referrals to strictly "screening" referrals (4,10). Patients with high-risk signs or symptoms, such as frank rectal bleeding or weight loss, were routed to definitive referrals through a more urgent process. ANHC also used immunochemical FOBT (iFOBT) kits rather than the more common and less expensive guaiac-based tests in hopes that the false-positive rate might be reduced, reasoning that the up-front investment in specificity would reduce the burden on the volunteer gastroenterologists.

**Table:** 

The first step is to determine whether the person is low-income uninsured. If no, then the person is referred through usual channels via the Referral Coordinator. If yes, then the person’s age determines the next step.
If the person is aged less than 50 years, then their level of risk is determined (high risk or intermediate risk). After a FOBT, high-risk people with either a positive or a negative test receive APA referral even without gastrointestinal symptoms. Intermediate-risk persons with a positive FOBT receive a referral for a colonoscopy. Intermediate-risk persons with a negative FOBT are recommended to receive a repeat FOBT in 6 months. If that test is positive, the person receives a referral for a colonoscopy. If that test is negative, then the process is ended and the person should consider a flexible sigmoidoscopy.
If the person is aged 50 years or older, a screening FOBT with a negative result ends the process and the person should consider a flexible sigmoidoscopy. If the screening test is positive, the person receives a referral for a colonoscopy. If the person is at intermediate risk, an FOBT with either a negative or positive test results in a referral for a colonoscopy. If the person is at high risk, an FOBT with either a negative or positive test results in a referral to Anchorage Project Access even if the person has no gastrointestinal symptoms.

Early in the program, ANHC tried to train its medical staff to provide screening by flexible sigmoidoscopy. However, it was difficult for staff to get enough training on practice patients and, once trained, to find time to do procedures among competing clinical demands. Because of these issues, the focus of the program shifted away from in-house flexible sigmoidoscopy and toward use of iFOBTs with positive follow-up colonoscopy referrals at participating private medical practices.

Initially, RFL provided funding only for the direct screening services: iFOBT kits and colonoscopy reimbursements. ANHC medical staff provided in-kind support for most of the project management, coordination, and referrals. However, this approach was burdensome to staff and did not lead to the desired increase in screening. In 2008, RFL agreed to fund a part-time staff position at ANHC for a screening care coordinator. After the creation of this position, screening numbers under the program increased substantially.

### Engaging the patient community

The screening program design was based on ANHC's previous vaccination outreach campaigns. Signs were placed throughout the clinic and in examination rooms, patient flyers were sent, and information was posted on the ANHC website. Holman and the medical director appeared with a private gastroenterologist on a local health care talk radio show to promote the screening program.

ANHC patients aged 50 to 70 years are sent an annual postcard during Colorectal Cancer Awareness Month (March) inviting them to pick up a free iFOBT kit at the clinic. Patients are identified as due for screening by using the ANHC electronic health record system or are referred to the program by ANHC providers; patients also may request the screening directly. Patients who bring back their completed iFOBT receive a $5 grocery store gift card. ANHC staff analyze the iFOBTs in an on-site laboratory.

The ANHC screening care coordinator contacts all patients with positive iFOBTs to come in to ANHC for follow-up according to a flow chart based on recommendations from the USPSTF and the American Cancer Society (Figure 2). Patients who have insurance are referred to private endoscopy centers for follow-up. Patients without insurance are invited to participate in the RFL-funded colonoscopy program. For these patients, the ANHC screening care coordinator reviews the patient's medical history and goes over the colonoscopy preparation and procedure instructions with the patient. The screening care coordinator then schedules the patient for a colonoscopy with 1 of the participating gastroenterologists at a private endoscopy center.

### Payments and reimbursements

After the colonoscopy appointment, the endoscopy center submits a payment authorization voucher to ANHC for reimbursement. The payment is $700, which covers about one-third of the overhead expense associated with the colonoscopy procedure (no physician fees are included). Under Alaska's Good Samaritan Law, because the providers waive their physician fees, they are immune from civil damages resulting from their services, with certain exceptions (eg, gross negligence) (11). Two laboratories process all of the pathology samples. The laboratories provide this service at a discounted flat rate of $75 per sample. RFL funds pay for the purchase and processing of the iFOBT kits, mailing and postage, patient incentives, and the salary of the part-time ANHC screening care coordinator. Should patients need further treatment, including further testing, repeat procedures, surgery, or hospitalization, payment is worked out by using various funding mechanisms.

## Outcomes

### Medical community

As a result of the efforts to engage the medical community, 16 doctors, 4 medical practices, and 2 laboratories agreed to provide services to ANHC patients identified through the CRC screening program ( Table). One gastroenterologist also serves as a champion for the program by directly encouraging other colonoscopy providers to participate in the program.

Barriers to the program included medical practice managers who were concerned about increased workload due to participation and about patient follow-up and care. Furthermore, some providers were concerned that if they initiated charity care they wouldn't be able to discontinue it. There is also the financial challenge of sustainability, which potentially limits the program in duration and scope since it depends on the annual fundraising capability of a nonprofit organization.

### Patient community

The program began outreach efforts to patients in December 2007 with $35,000 in funding from RFL; in 2008, RFL contributed $60,000. In the first year, 549 iFOBT kits were given to patients and 396 (72%) were returned (Table). The mean age of patients was 56 years (range, 40-66 y). A total of 24% were positive for fecal occult blood, and 7 uninsured patients received follow-up colonoscopies. No advanced polyps (tubular or more severe) were found during the exams. In 2008, numbers were similar to 2007 for kits distributed and returned. Of 16 uninsured patients who got follow-up colonoscopies, 2 had tubular adenomas and 1 had CRC diagnosed and treated. In 2009, a total of 1,390 iFOBT kits were given to patients and 680 (49%) were returned. Of those, 24% were positive for fecal occult blood. Of 57 colonoscopies completed, 9 were for patients who had tubular adenomas detected and removed. In the first quarter of 2010, a total of 23 colonoscopies were completed, and no advanced polyps or cancers were detected.

Challenges that patients face to participating in the program include language barriers and difficult living situations. There is a substantial time commitment required of patients who undergo a colonoscopy, including preparation, travel, waiting, procedure time, and onsite recovery (12). Many ANHC patients require translation for the colonoscopy instructions, for which ANHC uses a contract service that provides on-call language interpretation. This service is expensive but necessary to adequately prepare patients for their procedure. ANHC also creates some program materials in Spanish to facilitate patient comprehension. Colonoscopy preparation includes the use of strong laxatives the day before the procedure, which is difficult for homeless patients or those living in halfway or group housing. Some patients have difficulties finding an escort to take them home, which is required because of the sedatives administered during the procedure. Despite these potential barriers, 93% of ANHC patients scheduled for colonoscopies completed the procedure, which shows the high level of interest and dedication of these patients to accessing CRC screening when offered.

## Interpretation

This program provides a model for leveraging scarce resources to enable CRC screening for a low-income and underinsured and uninsured population. The program works by drawing on various partners: a fundraising organization, a community health center, and members of the medical community (specialty care including endoscopy and gastroenterology, and pathology services). These partnership and community engagement efforts have proved highly successful in increasing CRC screening in this underserved population.

This program was successful at getting low-income underinsured and uninsured patients screened for CRC because of several factors. The first is the persistence and leadership of Holman, who envisioned how a fundraising organization could work collaboratively with a primary care center by providing seed money for the initiation of a CRC screening program. The second is the trust built between ANHC and the community that it serves, which allowed the program to target services directly to patients in need of screening, resulting in a high iFOBT return rate by patients. Third is that by engaging the medical community, the program is able to tap into a willingness by providers, medical practices, and pathology groups to provide pro bono or heavily discounted CRC screening services. Lastly, the presence of a dedicated screening care coordinator based at ANHC is a necessary component of the program; this person is a liaison between the medical community members donating their services and patients in need of screening. The return rate for the iFOBT samples was 61%, which is higher than that reported in other published studies (13-15). We believe that the presence of the screening care coordinator, the support from other ANHC staff, and the small patient incentive help explain this high rate of adherence.

Recommendations for those seeking to initiate this sort of program are to hire at least a part-time screening care coordinator to manage the patient scheduling, instructions, billing, and colonoscopy results in order to facilitate appropriate patient care and follow-up. Many safety-net medical practices experience frequent staff turnover; thus, it is important to keep ongoing contact with people to maintain the smooth functioning of the program. A key component of such collaboration is to promote clear communication between all the groups involved: the physicians, the pathology services, the primary care facility, and the funders. Having memoranda of agreement in place is important, as is having a clear scope of work for all participating people and organizations to avoid delays in program implementation. Continued efforts to develop partnerships and engage the medical community will increase CRC screening services available and ultimately help to decrease deaths from CRC.

## Figures and Tables

**Table:** Patients Screened Through the Ride for Life Alaska and Anchorage Neighborhood Health Center Partnership, December 2007-April 2010

**Characteristic**	N (%)
**Status of iFOBTs**	
Given to patients	2,561 (100)
Returned	1,558 (61)
Positive	376 (24)
**Endoscopists participating in referral program**	16 (NA)
**Patients referred for program-funded colonoscopy**	111 (100)
Colonoscopies completed	103 (93)
Colonoscopies cancelled or refused	8 (7)
**Reason for referral^a^ (n = 101)**	
Positive iFOBT	51 (50)
Family history of CRC or polyps	27 (27)
Personal history of CRC or polyps	20 (20)
Screening	4 (4)
Other	3 (3)
**Sex (n = 102)**	
Men	50 (49)
Women	52 (51)
**Race (n = 101)**	
White	3 (3)
African American	15 (15)
Asian/Pacific Islander/American Indian	6 (6)
Unknown	77 (76)
**Results (n = 89)**	
Normal (including hyperplastic polyps)	73 (82)
Tubular adenoma or worse	15 (17)
Colorectal cancer	1 (1)

Abbreviations: iFOBT, immunochemical fecal occult blood test; NA, not applicable; CRC, colorectal cancer.

a Patients could have more than 1 reason for referral.
